# Dose de-escalation to the normal larynx using conformal radiotherapy reduces toxicity while maintaining oncologic outcome for T1/T2 glottic cancer

**DOI:** 10.1038/s41598-017-15974-6

**Published:** 2017-11-16

**Authors:** Jun Won Kim, Hyung Kwon Byeon, Hong-Shik Choi, Ik Jae Lee

**Affiliations:** 1 Department of Radiation Oncology, Gangnam Severance Hospital, Yonsei University College of Medicine, Seoul, Korea; 2 Department of Otorhinolaryngology, Head and Neck Cancer Clinic, Gangnam Severance Hospital, Yonsei University College of Medicine, Seoul, Korea

## Abstract

We evaluated the efficacy of dose de-escalation to the normal larynx using conformal radiotherapy (CRT) for T1/T2 glottic cancer. For conventional RT (2DRT, n = 38), the laryngeal box received a median equivalent dose in 2 Gy fractions (EQD2) of 66 Gy. For CRT (n = 42; 3D-CRT, 20; intensity-modulated RT, 22), clinical target volume (CTV)1 (gross tumor with a 3-mm margin) and CTV2 (laryngeal box) received median EQD2s of 66.6 Gy and 52.2 Gy, respectively. With a 71-month median follow-up, five-year local control and overall survival rates for 2DRT vs. CRT were 88.1% vs. 95.1% (p = 0.405) and 94.7% vs. 90.7% (p = 0.102), respectively. Grade 2 and 3 pharyngitis rates were 52.6% and 5.3% for 2DRT vs. 35.7% and 2.4% for CRT (p = 0.121). Grade 2 and 3 dermatitis rates were 42.1% and 2.6% for 2DRT vs. 35.7% and 0% for CRT (p = 0.013). The maximum phonation time increased from 12.1 ± 7.1 to 14.0 ± 6.6 seconds after 2DRT (p = 0.375) and from 12.0 ± 5.5 to 13.8 ± 10.1 seconds after CRT (p = 0.313). Fundamental frequency decreased from 150.6 ± 40.3 to 121.9 ± 30.2 Hz after 2DRT (p = 0.039) and from 138.5 ± 31.9 to 126.1 ± 23.7 Hz after CRT (p = 0.058). CRT can effectively de-escalate the normal larynx dose while maintaining oncologic outcome and voice quality.

## Introduction

Approximately two-thirds of laryngeal cancers arise in the glottic region, and 80–85% of those patients present with early–stage (T1–T2) disease^1^. Radiation therapy (RT) and endolaryngeal surgery are standard treatments for early-stage glottic cancers^2^. Intensity-modulated radiotherapy (IMRT) has been widely used to treat head and neck cancers and has yielded significant benefits in terms of reducing toxicity and improving the quality of life^3,4^. Uniquely, early glottic cancers (T1-T2N0) are associated with very low rates of cervical lymph node metastases (<3%)^5^. Unlike cancers in other sites of the head and neck, conventional RT is still widely used for early glottic cancers because the RT fields are relatively simple and only the larynx is treated without elective cervical lymph node irradiation^6^.

Conventional RT has yielded high cure rates for T1/T2 glottic cancer; however, patients receiving neck irradiation can experience acute toxicity such as radiation dermatitis, sore throat, and laryngeal edema^5^, as well as late toxicity such as carotid artery stenosis and an increased risk of stroke^7,8^. Recent attempts to utilize IMRT for early glottic cancer include carotid artery sparing for T1–2N0 cancers^9–12^ and single vocal cord (VC) irradiation for T1aN0 cancers^13,14^, with the intent to reduce doses to the carotid arteries and normal laryngeal tissues. Since 2007, we have used 3D conformal radiotherapy (3D-CRT) and IMRT to deliver definitive doses to the involved VC(s) while reducing doses to normal laryngeal tissues. We report and compare the outcomes and efficacy of our conformal RT protocol for dose de-escalation to the normal larynx with those of the conventional RT technique.

## Material and Methods

### Patients

All patients who were diagnosed with stage T1 or T2 squamous cell carcinoma of the glottic larynx, had clinically negative cervical lymph nodes (N0), and were treated with definitive RT between January 2004 and December 2013 were identified from our institutional database. All patients had undergone evaluations comprising medical history and physical examination, including indirect or flexible fiberoptic laryngoscopy, as well as staging computed tomography (CT) of the neck to rule out cervical lymph node involvement and extra-glottic spread. All suspicious lesions were biopsied, and slides were reviewed by head and neck pathology expert at our institution. Patients were divided into 2 groups according to RT technique: conventional RT (2DRT group) versus conformal RT with dose-reduction to the uninvolved larynx (CRT group). The study protocol conformed to the ethical guidelines of the 1975 Declaration of Helsinki, as revised in 1983, and was approved by the Institutional Review Board, Yonsei University Gangnam Severance Hospital (3-2016-0217), with a waiver of informed consent. This was a retrospective study for which all data were kept anonymous.

### Radiation techniques

**Figure 1:**
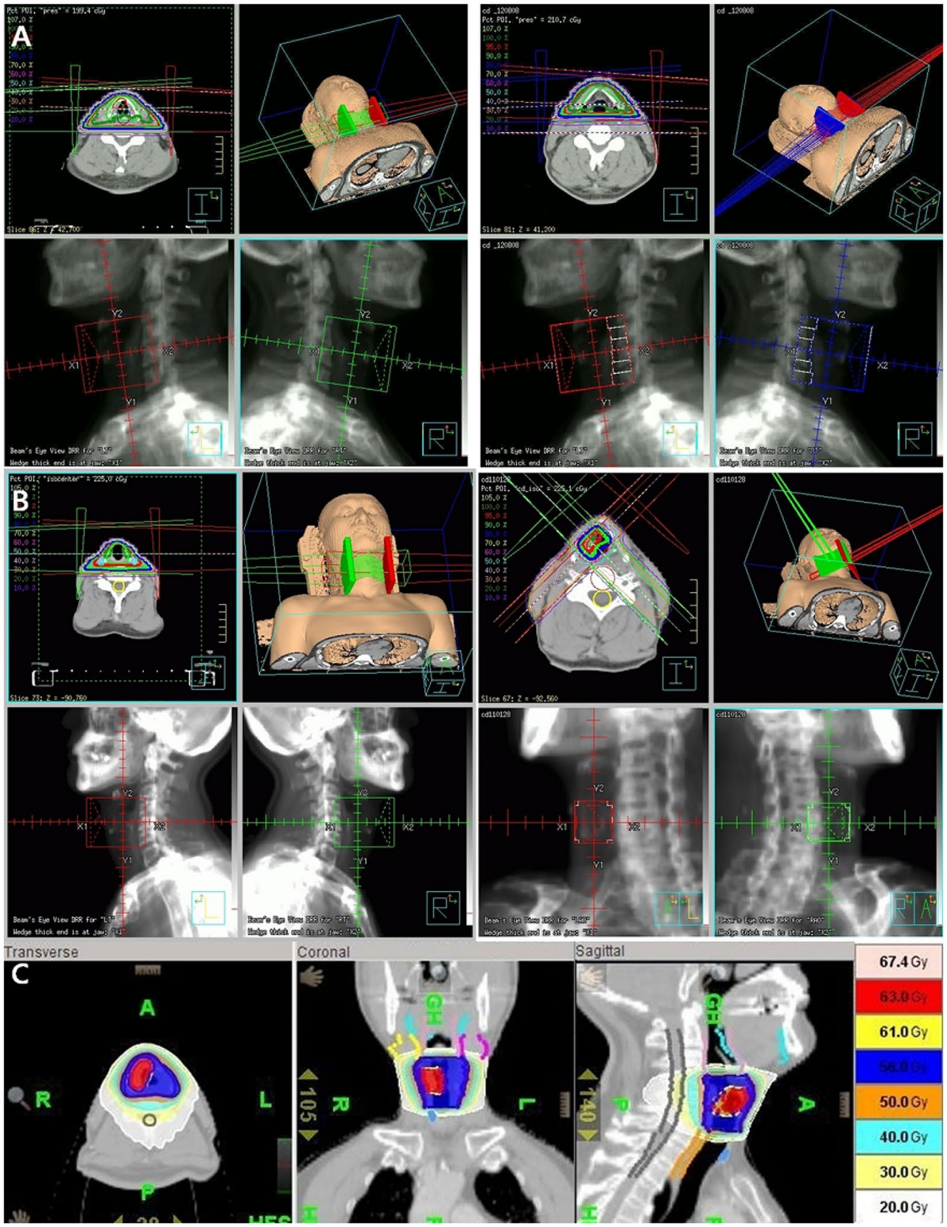
Radiotherapy (RT) plans for typical patients: conventional RT (2DRT) (**A**), 3-dimensional conformal RT (3D-CRT) with sequential boost (**B**), and simultaneous-integrated boost using intensity-modulated RT (SIB-IMRT) (**C**).

All patients received RT alone to the larynx without elective cervical lymph node irradiation. All patients underwent CT-based simulation with a 0.15–0.3 cm slice thickness following immobilization with a thermoplastic head and neck mask. For the 2DRT group, the laryngeal box was treated using parallel-opposed lateral fields. The borders were the top of the thyroid cartilage superiorly, the bottom of the cricoid inferiorly, 1-cm skin flash anteriorly, and 2-cm margin or anterior edge of the vertebral body posteriorly. The field size was typically 5 cm × 5 cm for T1N0 and 6 cm × 6 cm for T2N0 cases, with an extension to include the first tracheal ring (Fig. 1A). Patients who received CRT were treated with either a sequential boost using the 3D conformal RT (3D-CRT) technique or simultaneous-integrated boost using intensity-modulated RT (SIB-IMRT). The clinical target volume (CTV)1, which was equal to the gross tumor volume (GTV), encompassed all suspicious lesions identified from laryngoscopy and imaging studies; a 0.3-cm margin was added to define the planning target volume (PTV)1. CTV2 encompassed the laryngeal box and used the same borders described for 2DRT; a 0.5–1.0-cm margin was added to define PTV2. The sequential boost technique involved initial treatment with parallel-opposed lateral fields, as in 2DRT, and a cone-down to the PTV1 using 2 or 3 co-planar beams (Fig. 1B). Helical tomotherapy (Accuray, Madison, WI, USA) was used for SIB-IMRT (Fig. 1C). All plans were normalized so that ≥95% of the PTV received 100% of the prescription dose. Dose constraints included limiting the maximum dose in the PTV to 105% of the prescription and a soft constraint limiting the mean carotid artery dose to 30 Gy. Coverage was prioritized over carotid artery dose. The spinal cord dose was limited to 45 Gy at any point.

### Follow-up and statistical methods

Generally, patients were followed every 3 months for the first 2 years, every 6 months up to 5 years after radiotherapy and yearly thereafter. All failures were pathologically confirmed. Patients underwent voice assessments and laryngeal stroboscopic examinations. Voice quality assessments included aerodynamic measures and acoustic analyses^15^. Acute toxicity was evaluated using the Common Terminology Criteria for Adverse Events, version 4^16^.

The 2 treatment groups were compared using chi-square tests to detect differences in proportions. Survival was calculated from the date of tissue diagnosis until death or the most recent follow-up date. Survival time was analyzed using the Kaplan–Meier method, and the log-rank test was used for the univariate analysis. The Cox proportional hazards model was used to assess independent predictors of survival. Preoperative and postoperative results for each voice parameter were statistically compared using a paired t-test. SPSS 20.0 software for Windows (SPSS, Chicago, IL, USA) was used for these analyses, and statistical significance was defined as a p-value < 0.05.

## Results

### Patient characteristics

A total of 86 consecutively treated patients were reviewed. After excluding 3 patients treated with re-irradiation and 3 patients with synchronous double primary cancers, 80 patients were eligible for analysis. Among these 80 patients, 38 belonged to the 2DRT group and 42 to the CRT group (Supplementary Fig. 1). The median age was 62 years (range: 39–90 years), and most of the patients were men (96.2%) and mostly presented with T1–stage disease (56.3%); 30% of patients had anterior commissure involvement. The patient characteristics were well-balanced between the groups, except for a higher proportion of patients with a smoking-free interval ≥10 years in the CRT group (p = 0.015) (Table 1). Among patients in the 2DRT group, the laryngeal box was treated with a median equivalent dose in 2 Gy fractions (EQD2) of 66 Gy (range: 60–71 Gy). Among these patients, 19 (50%) underwent adaptive planning using a posterior pharyngeal block at a median dose of 50 Gy (range: 36–54 Gy) (Fig. 1A). In the CRT group, 20 patients received a sequential boost using 3D-CRT (Fig. 1B), and 22 patients received SIB-IMRT (Fig. 1C). Median EQD2s of 66.6 Gy (range: 64.3–68.9 Gy) to PTV1 and 52.2 Gy (range: 36.8–61 Gy) to PTV2, respectively, were prescribed.

**Table 1:** Patient Characteristics.

Characteristics	Total (n = 80)	2DRT (n = 38)	CRT (n = 42)	P value
Age				0.469
≤60 years	33 (41.3%)	15 (39.5%)	18 (42.9%)	
>60 years	47 (58.7%)	23 (60.5%)	24 (57.1%)	
Sex				0.538
Male	77 (96.2%)	37 (97.4%)	40 (95.2%)	
Female	3 (3.8%)	1 (2.6%)	2 (4.8%)	
T staging				0.487
Tis	7 (8.8%)	3 (7.9%)	4 (9.5%)	
T1a	47 (58.7%)	24 (63.2%)	23 (54.7%)	
T1b	6 (7.5%)	4 (10.5%)	2 (4.8%)	
T2	20 (25.0%)	7 (18.4%)	13 (31.0%)	
Smoking-free				0.015
≥10 years	32 (40.0%)	10 (26.3%)	22 (52.4%%)	
<10 year	48 (60.0%)	28 (73.7%)	20 (47.6%)	
Ant. Commissure				0.331
Not involved	56 (70.0%)	28 (73.7%)	28 (66.7%)	
Involved	24 (30.0%)	10 (26.3%)	14 (33.3%)	
Diagnosis				0.299
Biopsy	58 (72.5%)	26 (68.4%)	32 (76.2%)	
LMS	22 (27.5%)	12 (31.6%)	10 (23.8%)	
Total EQD2				0.282
≥66.6 Gy	50 (62.5%)	22 (57.9%)	28 (66.7%)	
<66.6 Gy	30 (37.5%)	16 (42.1%)	14 (33.3%)	

*Abbreviations:* LMS = laryngeal microscopic surgery; EQD2 = equivalent dose in 2 Gy fractions.

### Treatment outcomes

**Figure 2:**
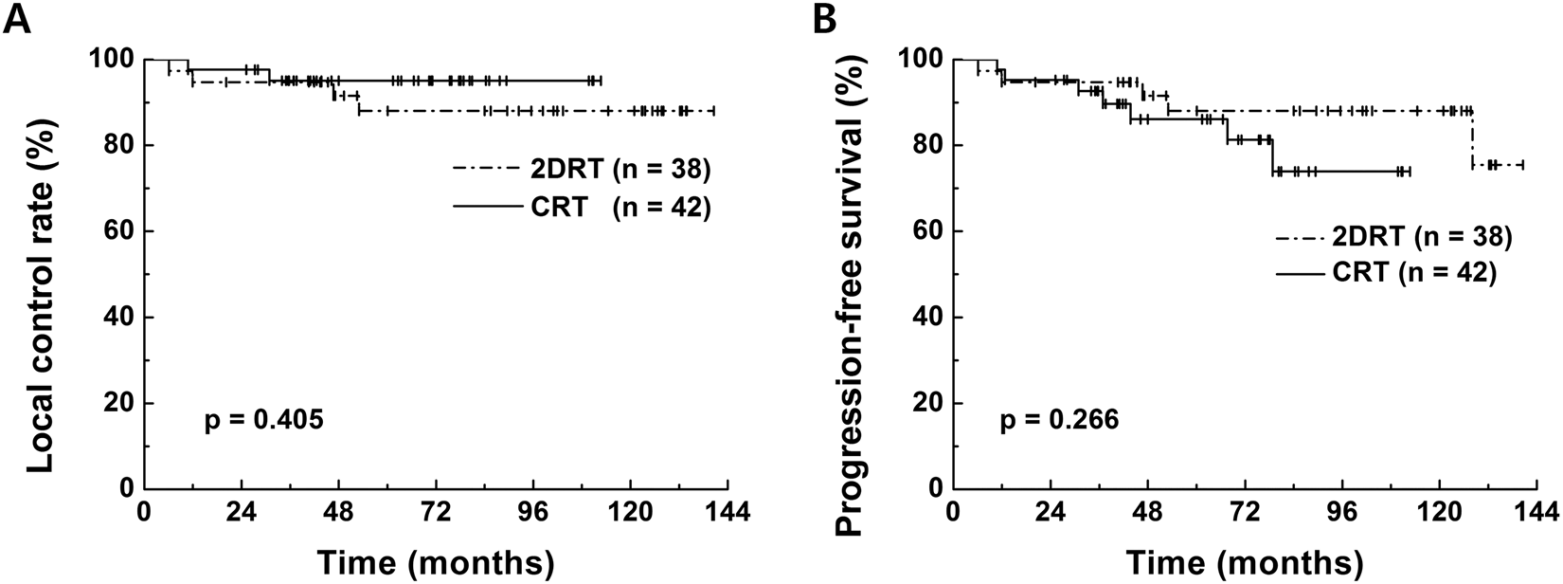
Comparison of treatment outcomes between the conventional radiotherapy (2DRT) and conformal RT (CRT) groups: local control rates (**A**) and progression-free survival (**B**).

The median follow-up was 71 months (range: 17–141 months; 2DRT, 97 months; CRT, 65 months). Seven patients experienced treatment failures. Two local, 1 local and regional, and 1 synchronous local, regional, and distant failure were reported in the 2DRT group; 2 local and 1 regional failures were reported in the CRT group. A detailed assessment of the failure sites is shown in Table 2. Among the 6 patients who experienced local failures, 1 had been treated with 3D-CRT with a sequential boost to a T2 tumor involving the entire right VC and had experienced recurrence in the contralateral true VC at 31 months after treatment. The 5-year local control (LC), progression-free survival (PFS), and overall survival (OS) rates for the 2DRT vs. CRT groups were 88.1% vs. 95.1% (p = 0.405) (Fig. 2A), 88.1% vs. 86.1% (p = 0.266) (Fig. 2B), and 94.7% vs. 90.7% (p = 0.102), respectively. In the CRT group, 3D-CRT with a sequential boost (n = 20) yielded 5-year LC, PFS, and OS rates of 95.0%, 80.0%, and 90.0%, respectively, and these values did not significantly differ from those of other RT modalities.

**Table 2:** Assessment of failure sites.

Age/Sex	Stage	Site	AC	Smoke free	RT	EQD2 (Gy)	Fail site	LF site (recur stage)	PFS (m)
85/M	T1a/Rt	A-M	no	current	2D	64.0	L	Ipsi ant-VC (rT1a)	6.1
61/M	T1a/Lt	Entire	no	3yrs	2D	64.0	L	Ipsi entire VC (rT1a)	53.0
48/M	Tis/Lt	A1/3	yes	4yrs	2D	64.0	L/R/D	Ipsi thyroid (rT4a)	46.7
69/M	T1b/Bl	Entire	no	7yrs	2D	66.0	L/R	Ipsi VC/thyroid (rT4a)	11.9
60/M	T2/Rt	Entire	yes	current	3DCRT	68.9	L	Cont mid-VC (rT1a)	30.9
50/M	T2/Rt	A-M	no	20yrs	3DCRT	68.9	R	None	12.7
62/M	T2/Rt	P1/3	no	current	IMRT	68.9	L/R/D	Ipsi post-VC (rT2)	10.8

*Abbreviations:* Rt = right; Lt = left; Bl = bilateral; A-M = anterior to middle cord; A1/3 = anterior 1/3; P1/3 = posterior 1/3; AC = anterior commissure involvement; EQD2 = equivalent dose in 2 Gy fractions; L = local; R = regional; L/R = local and regional; L/R/D = local and regional and distant; LF = local failure; Ipsi = ipsilateral; VC = vocal cord; Cont = contralateral; PFS = disease-free survival; m = months.

### Prognostic factor analysis

Age (≤60 vs. >60 years), T stage (Tis/T1 vs. T2), anterior commissure involvement, smoking-free interval (≥10 vs. <10 years), EQD2 (≥66.6 vs. <66.6 Gy), and RT technique (CRT vs. 2DRT) were included in the univariate analysis; of these, only a smoking-free interval <10 years associated significantly with decreased LC (p = 0.041). We were unable to analyze the association between the smoking-free interval and LC rate in a multivariate analysis because none of the patients with a smoking-free interval ≥10 years experienced a recurrence or was censored (Table 3).

**Table 3:** Prognostic factors affecting local control rate.

Variables	No patients (%)	Univariate			Multivariate		
		5-yr LFFS (%)	95%CI	P	RR	95%CI	P
Age							
≤60	33 (41.3)	92.7	83–103	0.640			
>60	47 (58.7)	90.1	81–100				
T stage							
Tis/T1	60 (75.0)	91.6	84–100	0.651			
T2	20 (25.0)	89.7	76–103				
Ant commissure							
Not involved	56 (70.0)	91.9	84–100	0.851			
Involved	24 (30.0)	89.3	75–104				
Smoking-free							
≥10 years	32 (40.0)	100	n/a	0.041	n/a	n/a	n/a
<10 years	48 (60.0)	85.6	75–96				
EQD2							
≥66.6 Gy	48 (60.0)	94.0	87–101	0.335	0.506	0.10–2.60	0.415
<66.6 Gy	32 (40.0)	79.8	57–102				
RT technique							
CRT	42 (52.5)	95.1	88–102	0.405	1.156	0.21–6.47	0.869
2DRT	38 (47.5)	88.1	77–99				

*Abbreviations:* CI = confidence interval; RR = relative risk; EQD2 = equivalent dose in 2 Gy fractions; CRT = conformal radiotherapy; 2DRT = conventional radiotherapy.

### Toxicity and voice quality preservation

The incidence of radiation-induced acute toxicity according to treatment groups is summarized in Table 4. Rates of grade 2 and 3 pharyngitis were 52.6% and 5.3% for 2DRT vs. 35.7% and 2.4% for CRT (p = 0.121). Rates of grade 2 and 3 dermatitis were 42.1% and 2.6% for 2DRT vs. 35.7% and 0% for CRT (p = 0.013). In the CRT group, rates of grade 2 and 3 pharyngitis were 35.0% and 0% for 3D-CRT vs. 36.4% and 4.5% for IMRT (p = 0.127), and rates of grade 2 and 3 dermatitis were 50.0% and 0% for 3DCRT vs. 22.7% and 0% for IMRT (p = 0.001). Both pre-RT and delayed post-RT (>6 months) voice quality assessments were available for 40 patients (n = 14 for 2DRT and 26 for CRT), and voice qualities were compared between the 2 time points. The maximum phonation time (MPT) increased from 12.1 ± 6.1 seconds to 13.9 ± 9.0 seconds for all patients (p = 0.176), from 12.1 ± 7.1 seconds to 14.0 ± 6.6 seconds for the 2DRT group (p = 0.375), and from 12.0 ± 5.5 to 13.8 ± 10.1 seconds for the CRT group (p = 0.313). The average fundamental frequency (F0) decreased from 142.7 ± 35.0 Hz to 124.7 ± 25.9 Hz for all patients (p = 0.005), from 150.6 ± 40.3 Hz to 121.9 ± 30.2 Hz for 2DRT (p = 0.039), and from 138.5 ± 31.9 Hz to 126.1 ± 23.7 Hz for CRT (p = 0.058) (Table 5). In the CRT group, MPT increased from 13.5 ± 5.9 to 16.3 ± 12.8 seconds for the 3D-CRT group (p = 0.378) and from 10.8 ± 5.1 to 11.7 ± 7.0 seconds for the IMRT group (p = 0.654), and F0 decreased from 138.7 ± 32.7 Hz to 132.6 ± 29.9 Hz for 3D-CRT (p = 0.252) and from 138.3 ± 32.4 Hz to 120.6 ± 16.0 Hz for IMRT (p = 0.123).

**Table 4:** Acute toxicity.

		Total (n = 80)	2DRT (n = 38)	CRT (n = 42)	P value
Pharyngitis	Grade 0	4	0	4 (9.5%)	0.121
	Grade 1	38	16 (42.1%)	22 (52.4%)	
	Grade 2	35	20 (52.6%)	15 (35.7%)	
	Grade 3	3	2 (5.3%)	1 (2.4%)	
Dermatitis	Grade 0	21	4 (10.5%)	17 (40.5%)	0.013
	Grade 1	27	17 (44.7%)	10 (23.8%)	
	Grade 2	31	16 (42.1%)	15 (35.7%)	
	Grade 3	1	1 (2.6%)	0

**Table 5:** Changes in maximum phonation time and fundamental frequency (n = 40/80).

		Pre-RT	Post-RT ≥ 6 mo	P
MPT (sec)	All (n = 40/80)	12.1 ± 6.1	13.9 ± 9.0	0.176
	2DRT (n = 14/38)	12.1 ± 7.1	14.0 ± 6.6	0.375
	CRT (n = 26/42)	12.0 ± 5.5	13.8 ± 10.1	0.313
F0 (Hz)	All (n = 40/80)	142.7 ± 35.0	124.7 ± 25.9	0.005
	2DRT (n = 14/38)	150.6 ± 40.3	121.9 ± 30.2	0.039
	CRT (n = 26/42)	138.5 ± 31.9	126.1 ± 23.7	0.058

*Abbreviations:* MPT = maximum phonation time; F0 = average fundamental frequency.

## Discussion

Previous studies of conventional RT have reported 5-year LC rates of 84–95% for T1a and 50–85% for T2 glottic cancers^17^. In malignancies associated with long-term survival, such as early glottic cancer, the greatest opportunity for therapeutic advancement lies in the prevention of toxicity while maintaining excellent oncologic outcomes. The current study included patients with Tis (9%), T1 (56%), and T2 (25%) disease, and achieved 5-year LC and OS rates of 91.3% and 93.1%, respectively. These results were comparable to those of previous reports^17^. Attempts have been made to reduce normal tissue toxicity during the delivery of definitive RT for early glottic cancers. Several studies have demonstrated the role of IMRT in reducing the doses delivered to the carotid arteries^9,10,12^ and soft tissues of the neck^18^, although the long-term benefits of these efforts remain to be established. Apparent concerns regarding increased local failures emerge when only a single VC is irradiated, although most studies consider selected patients with T1a cancers to be eligible for such treatment. For example, Levendag *et al*. reported a 5-year LC rate of 93% after single VC irradiation in 164 patients with T1a cancers^14^. Despite the very limited clinical experience, single VC irradiation seems feasible in selected T1a patients.

In the current study, the treatment scheme applied to the CRT group was somewhat unique in that the involved VC received the full dose of 67 Gy EQD2, whereas the remainder of the larynx received a reduced dose of 52 Gy EQD2 with the intent to reduce toxicity to the normal larynx while maintaining LC. The 5-year LC rates were 95.1% for the CRT group and 88.1% for the 2DRT group, and demonstrate the efficacy of our CRT protocol. Unexpectedly, the LC rate with 2DRT was lower than with CRT. We demonstrated a significant association between a longer smoking-free interval and LC, and noted that the CRT group had a significantly higher proportion of patients with a smoking-free interval ≥10 years (Table 1); accordingly, the higher LC rate in the CRT group might be attributable to this inter-group difference in the smoking-free interval.

A recurrence in the contralateral true VC was reported in 1 patient in the CRT group. This patient exhibited strong risk factors for recurrence, including T2 stage disease, a tumor involving the entire VC and anterior commissure, and a status as a current smoker at the time of diagnosis. Interestingly, 2DRT resulted in local failures only in patients with Tis/T1 glottic cancers, whereas CRT resulted in local failures only in patients with T2 glottic cancers (Table 2). In the American Joint Committee on Cancer staging manual, 7^th^ edition^19^, a designation of T2 glottic cancer requires extension of the disease to the supraglottis and/or subglottis; however, such tumor extension is not readily detectable on CT images, thus increasing the difficulty of accurate target delineation. Possibly, our current definition of PTV1 as gross disease with a 3-mm margin might not have provided adequate tumor coverage in T2 cases. A study conducted at Princess Margaret Hospital compared conventional RT (n = 48) and normal tissue-sparing IMRT (n = 50) exclusively for T2 glottic cancer. For IMRT, CTV60 was defined as a 0.5–1.0 cm expansion on the GTV, and CTV50 was variably defined to include a further expansion of 0.5–2.0 cm on the CTV60. In 17 of the 50 IMRT cases, the CTV50 was expanded to include the whole larynx; however, the 3-year local recurrence rate was higher when compared to that of conventional RT (32% vs. 20%, p = 0.54). In all cases, the local relapse sites included the supraglottis 64% and subglottis 88%^20^. Based on these observations, we have modified our scheme of target delineation for T2 glottic cancer as follows: PTV1 is defined by adding 0.3-cm margins around all suspicious lesions (GTV) identified via laryngoscopy and imaging studies; PTV2 is equal to CTV and encompasses the entire larynx, including the both the anterior and posterior commissures and arytenoids; and PTV3 is defined as a 0.5–1-cm expansion of the PTV2. The prescribed doses to PTV1, PTV2, and PTV3 are 65.25 Gy, 58.0 Gy, and 49.3 Gy delivered over 29 fractions, respectively.

Although conventional RT provides high rates of LC for early glottic cancer, there remains room for improvement in terms of toxicity reduction. The incidence rates of grade 2 and 3 dermatitis were 42% and 1% with 2DRT vs. 36% and 0% with CRT (p = 0.013). There is a concern that IMRT increases the dose to the skin in head and neck cancer patients^6^. In our protocol, however, the skin is intentionally spared from target delineation unless the anterior commissure is involved, and the resultant incidence of dermatitis supports the efficacy of our CRT protocol with regard to reducing acute skin toxicity. Even in cases of grade 2 dermatitis, the involved skin area was limited to that adjacent to the anterior commissure, thus reducing discomfort and improving patient compliance with treatment. Despite the significant reduction in dermatitis, CRT did not result in significant reduction in the rates of grade 2 and 3 pharyngitis when compared with 2DRT (p = 0.121). At our institution, if the disease is well localized in the anterior half of the VC, a posterior pharyngeal block is often employed during 2DRT delivery to reduce the occurrence of odynophagia. In the current study, 50% of patients in the 2DRT group underwent treatment with adaptive plans for pharyngeal sparing, thus reducing the risk of pharyngitis.

Preservation of voice quality is a key factor when deciding treatment options for early glottic cancer. Previous studies have compared the functional outcomes, including voice quality, of transoral laser surgery and conventional RT^21–23^; however, to our knowledge, no studies have compared the functional outcomes of IMRT and conventional RT modalities. Unfortunately, routine assessments of voice quality before and after radiotherapy were not implemented at our institution until 2008; as a result, these data are only available for 50% of the total cohort, with an imbalance in data availability between the 2 treatment groups. Although the maximum phonation time (MPH) improved after both 2DRT and CRT, these improvements were not significant. The average fundamental frequency (F0) also improved after both treatment modalities; this difference was statistically significant in the 2DRT group (p = 0.039) and indicated a trend of improvement in the CRT group (p = 0.058). Although many confounding factors influence the voice quality beyond 6 months after RT, the long-term changes in voice quality in the treatment groups appeared comparable. A further report will follow, as the voice quality data are being accumulated using standardized assessment procedures.

This study has several limitations. This was a retrospective study, and thus confounding factors between the CRT and 2DRT groups might have affected the results. Indeed, the CRT group had a higher proportion of patients with a smoking-free interval ≥10 years, and therefore had a higher (although not statistically significant) LC rate when compared with the 2DRT group. Both groups, however, exhibited LC rates comparable to those in previous reports, thus demonstrating the efficacy of our CRT protocol. The CRT group comprised patients treated with 2 different RT modalities: 3D-CRT with sequential boost and SIB-IMRT. The Korean National Health Insurance began reimbursing costs related to IMRT for head and neck cancer in 2011, and since then, we have employed SIB-IMRT for all early glottic cancer cases. Although the numbers were too small for statistical comparison, the treatment outcomes of 3D-CRT (95.0% LC and 90.0% OS) were comparable to those of other RT modalities in the current study. Furthermore, the retrospective nature meant that toxicity assessments were not performed at regular intervals, which somewhat reduced the validity of our toxicity comparison between the 2 treatment groups. Nevertheless, several studies have previously shown the dosimetric advantages of IMRT in terms of reducing the doses to laryngeal soft tissues and carotid arteries. We are currently following up IMRT-treated patients in the context of a prospective observational study, and an additional report will follow.

## Conclusion

CRT can be used to effectively de-escalate the dose to the normal larynx in the context of early glottic cancer treatment, resulting in reduced toxicity while maintaining oncologic outcomes and voice quality. The use of IMRT for maintaining effective tumor control of T2 glottic cancer requires attention to the extent of the disease in target delineation.

## Electronic supplementary material

Supplementary Figure 1

## References

[1] Ferlay J (2010). Estimates of worldwide burden of cancer in 2008: GLOBOCAN 2008. Int J Cancer.

[2] Pfister DG (2014). Head and neck cancers, Version 2.2014. Clinical practice guidelines in oncology. J Natl Compr Canc Netw.

[3] Marta GN (2014). Intensity-modulated radiation therapy for head and neck cancer: systematic review and meta-analysis. Radiother Oncol.

[4] Gregoire V, Langendijk JA, Nuyts S (2015). Advances in Radiotherapy for Head and Neck Cancer. J Clin Oncol.

[5] Mendenhall WM, Amdur RJ, Morris CG, Hinerman RW (2001). T1-T2N0 squamous cell carcinoma of the glottic larynx treated with radiation therapy. J Clin Oncol.

[6] Feigenberg SJ, Lango M, Nicolaou N, Ridge JA (2007). Intensity-modulated radiotherapy for early larynx cancer: is there a role?. Int J Radiat Oncol Biol Phys.

[7] Swisher-McClure S (2014). Risk of fatal cerebrovascular accidents after external beam radiation therapy for early-stage glottic laryngeal cancer. Head Neck.

[8] Muzaffar K, Collins SL, Labropoulos N, Baker WH (2000). A prospective study of the effects of irradiation on the carotid artery. Laryngoscope.

[9] Zumsteg ZS (2015). Carotid sparing intensity-modulated radiation therapy achieves comparable locoregional control to conventional radiotherapy in T1-2N0 laryngeal carcinoma. Oral Oncol.

[10] Chera BS, Amdur RJ, Morris CG, Mendenhall WM (2010). Carotid-sparing intensity-modulated radiotherapy for early-stage squamous cell carcinoma of the true vocal cord. Int J Radiat Oncol Biol Phys.

[11] Gomez D, Cahlon O, Mechalakos J, Lee N (2010). An investigation of intensity-modulated radiation therapy versus conventional two-dimensional and 3D-conformal radiation therapy for early stage larynx cancer. Radiat Oncol.

[12] Choi HS (2016). Carotid sparing intensity modulated radiotherapy on early glottic cancer: preliminary study. Radiat Oncol J.

[13] Al-Mamgani A (2015). Single Vocal Cord Irradiation: Image Guided Intensity Modulated Hypofractionated Radiation Therapy for T1a Glottic Cancer: Early Clinical Results. Int J Radiat Oncol Biol Phys.

[14] Levendag PC (2011). Single vocal cord irradiation: a competitive treatment strategy in early glottic cancer. Radiother Oncol.

[15] Byeon HK (2015). Treatment of Hemorrhagic Vocal Polyps by Pulsed Dye Laser-Assisted Laryngomicrosurgery. Biomed Res Int.

[16] U.S. Department of Health and Human Services, N. I. o. H., National Cancer Institute., *Common Terminology Criteria for Adverse Events (CTCAE) version4.03* (2010). Available at: http://evs.nci.nih.gov/ftp1/CTCAE/CTCAE_4.03_2010-06-14_QuickReference_5x7.pdf. (Accessed: August 6, 2016).

[17] Hartl DM (2011). Evidence-based review of treatment options for patients with glottic cancer. Head Neck.

[18] Penagaricano JA, Ratanatharathorn V, Papanikolaou N, Yan Y (2004). Intensity-modulated radiation therapy reduces the dose to normal tissue in T2N0M0 squamous cell carcinoma of the glottic larynx. Med Dosim.

[19] Edge, S. B., B. D., Compton, C. C., Fritz, A. G., Greene, F. L., Trotti, A. & editors. *AJCC cancer staging manual 7th edn* (Springer, 2010).

[20] Tiong AC (2011). Outcomes for T2N0M0 Glottic Squamous Cell Carcinoma Treated with IMRT Compared with Conventional Parallel Opposed Fields. Int J Radiat Oncol Biol Phys.

[21] Aaltonen LM (2014). Voice quality after treatment of early vocal cord cancer: a randomized trial comparing laser surgery with radiation therapy. Int J Radiat Oncol Biol Phys.

[22] Spielmann PM, Majumdar S, Morton RP (2010). Quality of life and functional outcomes in the management of early glottic carcinoma: a systematic review of studies comparing radiotherapy and transoral laser microsurgery. Clin Otolaryngol.

[23] Abdurehim Y (2012). Transoral laser surgery versus radiotherapy: systematic review and meta-analysis for treatment options of T1a glottic cancer. Head & neck.

