# Publisher Correction: Dose de-escalation to the normal larynx using conformal radiotherapy reduces toxicity while maintaining oncologic outcome for T1/T2 glottic cancer

**DOI:** 10.1038/s41598-018-26659-z

**Published:** 2018-06-07

**Authors:** Jun Won Kim, Hyung Kwon Byeon, Hong-Shik Choi, Ik Jae Lee

**Affiliations:** 1 Department of Radiation Oncology, Gangnam Severance Hospital, Yonsei University College of Medicine, Seoul, Korea; 2 Department of Otorhinolaryngology, Head and Neck Cancer Clinic, Gangnam Severance Hospital, Yonsei University College of Medicine, Seoul, Korea

Correction to: *Scientific Reports* 10.1038/s41598-017-15974-6, published online 16 November 2017

In the original HTML version of this Article, Hong-Shik Choi, and not Ik Jae Lee, was listed as the corresponding author. Correspondence and request for materials should be addressed to Dr. Ik Jae Lee at ikjae412@yuhs.ac. This has now been corrected in the HTML version of the Article; the PDF version was correct from the time of publication.

