# Supplementary material for: Dose de-escalation to the normal larynx using conformal radiotherapy reduces toxicity while maintaining oncologic outcome for T1/T2 glottic cancer
Source: Sci Rep. 2017 Nov 16;7:15732. doi: 10.1038/s41598-017-15974-6 (PMC5691141; doi:10.1038/s41598-017-15974-6)

Dose de-escalation to the normal larynx using conformal radiotherapy reduces toxicity while maintaining oncologic outcome for T1/T2 glottic cancer

Jun Won Kim, MD[a,*], Hyung Kwon Byeon, MD[b], Hong-Shik Choi, MD[b], Ik Jae Lee, MD[a]

Departments of [a]Radiation Oncology and [b]Otorhinolaryngology, Head and Neck Cancer Clinic, Gangnam Severance Hospital, Yonsei University College of Medicine, Seoul, Korea

*Corresponding author: Ik Jae Lee, M.D., Ph.D.

Department of Radiation Oncology, Gangnam Severance Hospital, Yonsei University College of Medicine, 211 Eonju-ro, Gangnam-gu, Seoul 06273, Republic of Korea

Tel: 82-2-2019-3158; Fax: 82-2-2019-4855; E-mail: ikjae412@yuhs.ac

The abstract of this study was accepted for poster viewing at the 58th Annual Meeting of the American Society for Radiation Oncology (ASTRO), Boston, MA, September 2016.

Supplementary Fig. 1 Eligibility criteria

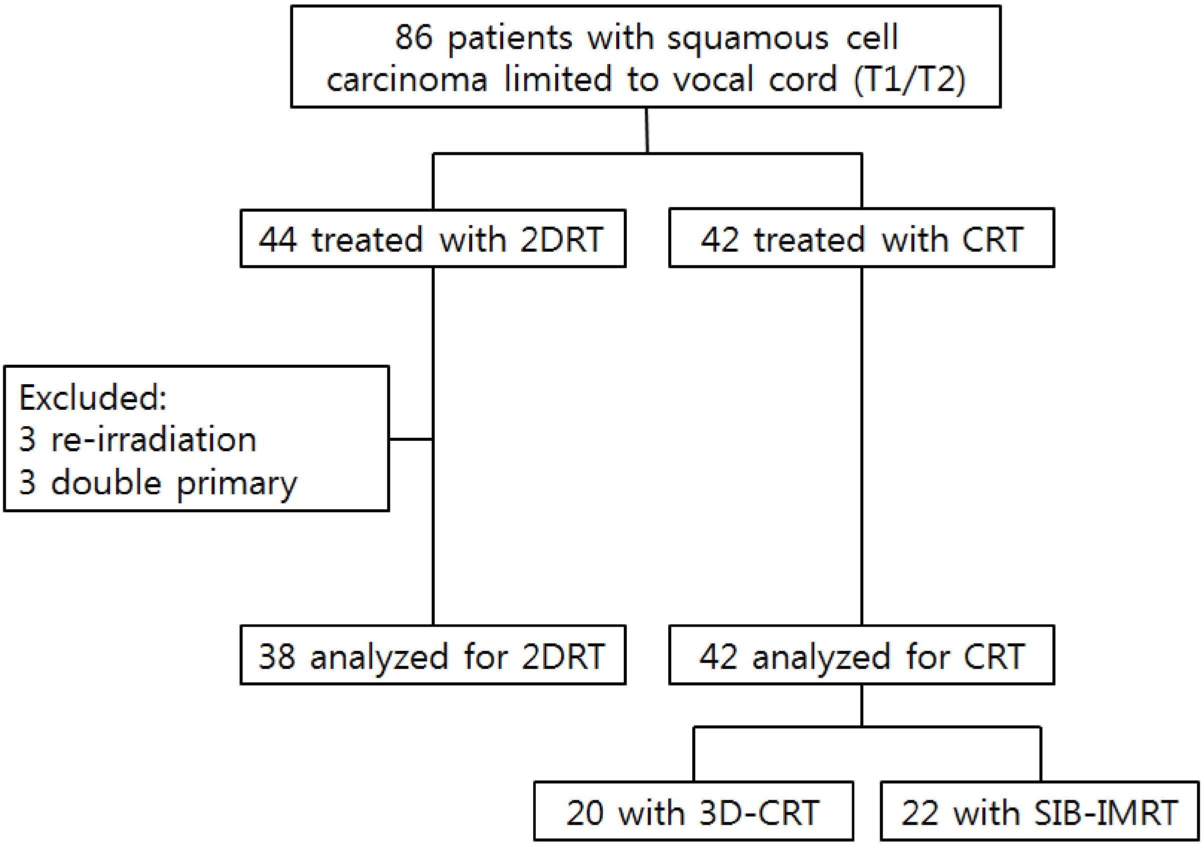

Supplement: Supplementary file 1 — Supplementary Figure 1 [file 41598_2017_15974_MOESM1_ESM.pdf]
